# Multiple Linear Discriminant Models for Extracting Salient Characteristic Patterns in Capsule Endoscopy Images for Multi-Disease Detection

**DOI:** 10.1109/JTEHM.2020.2964666

**Published:** 2020-01-17

**Authors:** Amit Kumar Kundu, Shaikh Anowarul Fattah, Khan A. Wahid

**Affiliations:** 1 Department of Electrical and Electronic EngineeringBangladesh University of Engineering and Technology 61750 Dhaka1205Bangladesh; 2 Department of Electrical and Computer EngineeringUniversity of Saskatchewan 7235 SaskatoonSKS7N 5A9Canada

**Keywords:** Capsule endoscopy, linear discriminant analysis, gastrointestinal disease detection, probability density function model, support vector machine

## Abstract

Background: Computer-aided disease detection schemes from wireless capsule endoscopy (WCE) videos have received great attention by the researchers for reducing physicians’ burden due to the time-consuming and risky manual review process. While single disease classification schemes are greatly dealt by the researchers in the past, developing a unified scheme which is capable of detecting multiple gastrointestinal (GI) diseases is very challenging due to the highly irregular behavior of diseased images in terms of color patterns. Method: In this paper, a computer-aided method is developed to detect multiple GI diseases from WCE videos utilizing linear discriminant analysis (LDA) based region of interest (ROI) separation scheme followed by a probabilistic model fitting approach. Commonly in training phase, as pixel-labeled images are available in small number, only the image-level annotations are used for detecting diseases in WCE images, whereas pixel-level knowledge, although a major source for learning the disease characteristics, is left unused. In view of learning the characteristic disease patterns from pixel-labeled images, a set of LDA models are trained which are later used to extract the salient ROI from WCE images both in training and testing stages. The intensity patterns of ROI are then modeled by a suitable probability distribution and the fitted parameters of the distribution are utilized as features in a supervised cascaded classification scheme. Results: For the purpose of validation of the proposed multi-disease detection scheme, a set of pixel-labeled images of bleeding, ulcer and tumor are used to extract the LDA models and then, a large WCE dataset is used for training and testing. A high level of accuracy is achieved even with a small number of pixel-labeled images. Conclusion: Therefore, the proposed scheme is expected to help physicians in reviewing a large number of WCE images to diagnose different GI diseases.

## Graphical abstract

An automatic multiple GI disease detection scheme is proposed in this paper based on PDF modeling of multiple linear discriminant analysis-based regions of interest from from wireless capsule endoscopy videos.

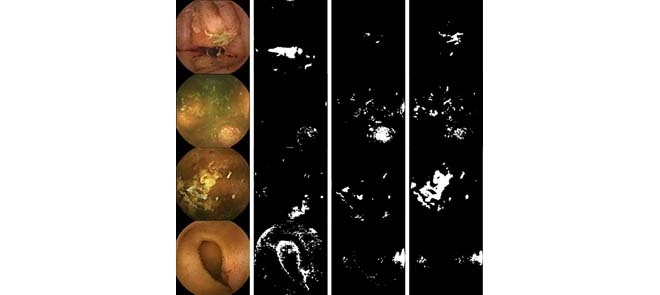

## I. Introduction

Diseases related to the gastrointestinal (GI) tract, such as bleeding, ulcer and tumor are a great threat to human health. Therefore, detecting and diagnosing GI diseases using the wireless capsule endoscopy (WCE) video technology have received great clinical importance [1]. However, manual inspection of long duration WCE video requires a huge amount of effort and preciseness by the physicians [2]. As a result, researchers became motivated to develop computer-aided methods to detect the GI diseases for reducing the burden of the physicians [1]. With the exception of a few, most research efforts are concentrated on dealing with detecting only one type of disease where bleeding, being the most common GI disease, has received the most attention [3]–[13]. There are a few schemes that focus on ulcer or tumor detection from WCE videos [14]–[21]. Various approaches those are used for the development of automatic bleeding detection methods are based on suspected blood indicator [3], statistical features [8], pixel intensity histogram-based features [5], [9], [22], block-based approaches [6], bag-of-words (BOW) based approach [7], salient-point based approaches, [12], [23] and deep learning architectures [10], [11]. Moreover, computer-aided ulcer and erosion detection methods are developed using convolutional neural network (CNN) based architecture [15], completed local binary pattern (LBP), and laplacian pyramid [14], and indexed image based approach [16]. On the other side, tumor recognition methods are developed using textural descriptors in inverse curvelet domain [17], discrete wavelet transform [18], uniform LBP [19], and Gabor filter-bank [20]. In [21], a stacked sparse autoencoder with image manifold constraint is proposed to detect polyps from WCE images. However, these classification schemes concentrate on only a single disease class. Limited works are reported that deal with multiple GI disease detection from WCE videos [24]–[26]. However, their performance is yet to be significantly improved. In [24], a saliency and adaptive locality-constrained linear coding (SALLC) is employed to encode the local block features for multiple GI disease detection. In [25], a multi-texture analysis based approach is proposed while in [26], a discriminatory joint-feature model is employed to classify multiple diseases in WCE images. In the available multiple GI disease detection methods, the entire WCE image is utilized for feature extraction, which may degrade quality of the feature when diseased portions are significantly small. One possible way is to capture preliminary suspected region of interest (ROI) before feature extraction. Here, precise ROI extraction ensures better feature quality for disease detection. In recent times, the deep learning-based schemes overcame the need for ROI segmentation for disease classification [15], [21]. However, in the case of WCE, ROI segmentation plays an important role for physicians. ROI segmentation of WCE images can provide rapid visualization of the diseased portions, and the physicians can examine the successive changes of diseased lesions in WCE videos. Therefore, different segmentation schemes are proposed to highlight particular types of diseased lesions. For example, deep learning-based schemes are proposed to segment bleeding in [27] using SegNet, to segment mucosa in [28] using CNN, to segment Angiodysplasia in [29] using CNN encoder-decoder architecture, Esophageal Cancer in [30] using Deeplabv3+ network, and red lesions in [10] using U-net. These deep methods require extensive training using a lot of images for region segmentation, and different types of deep models are trained for capturing different types of disease characteristics. Moreover, in addition to the region segmentation model, separate models are required to train for disease classification. Therefore, the major challenge is to propose a unified ROI extraction scheme from various diseases because of the highly irregular characteristic patterns in different diseases. On the other hand, the pixel-labeled images can be utilized as a major resource for learning the characteristic patterns of diseased pixels, even if these images are available only in a small amount. Hence, a computer-aided disease detection scheme, which can capture the salient patterns from any disease type through learning with the help of pixel-labeled images, is yet to be investigated.

The objective of this paper is show that with the help of multiple linear discriminant analysis (LDA) models, which are trained using a small number of pixel-labeled diseased images, effective ROIs can be obtained which in turn can extract the precise characteristic nature of diseases. At first, the LDA models are trained separately using a few pixel-labeled images of different diseases. Although pixel-labeled images are rarely utilized in the past, these images can be a major source of learning the disease patterns even if available in small number. The training ensures that the trained LDA models learn the nature of disease from the prior pixel-labeled images. Next, to extract the salient region of interest (ROI) in WCE images, the trained LDA models are applied. A characteristic probability density function (PDF) is fitted to model the intensity patterns of ROI. A consistent class representation along with a very low feature dimension is ensured because of such a PDF-fitting based scheme. Finally, an efficient hierarchical classification scheme is employed in this paper using a few binary SVM classifiers to detect multiple GI diseases from WCE images.

The contribution of this paper can be summarized as follows:

• This paper proposes a unified computer aided scheme which detects multiple GI diseases, like ulcer, tumor and bleeding from WCE images. Although there are only few works available which deal with multi-disease classification problem, their performance is yet to be improved.
• A certain portion of training images generally contain pixel labeling, where the diseased portions are marked by the expert physicians. However, most of the automatic disease detection schemes available in the literature do not utilize pixel-labeled images for disease detection. The pixel-labeled images can serve as a major source of information for learning about a particular disease, no matter even they are available for a small number. Therefore, in this paper, we propose to utilize the pixel-labeled images for ROI extraction from a WCE image.
• The existing works extract features from the entire WCE image which may degrade the performance of the scheme in cases where the diseased portion is really small. However, this paper proposes a preliminary ROI selection scheme irrespective of the type of disease and then features are extracted from the selected ROI.

## II. Proposed Multiple GI Disease Detection Scheme Based on Modeling of LDA Classified Region of Interest

**FIGURE 1.**
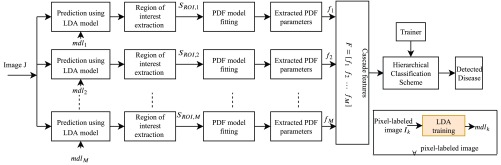
Block diagram of the proposed multi-disease detection method.

**FIGURE 2.**
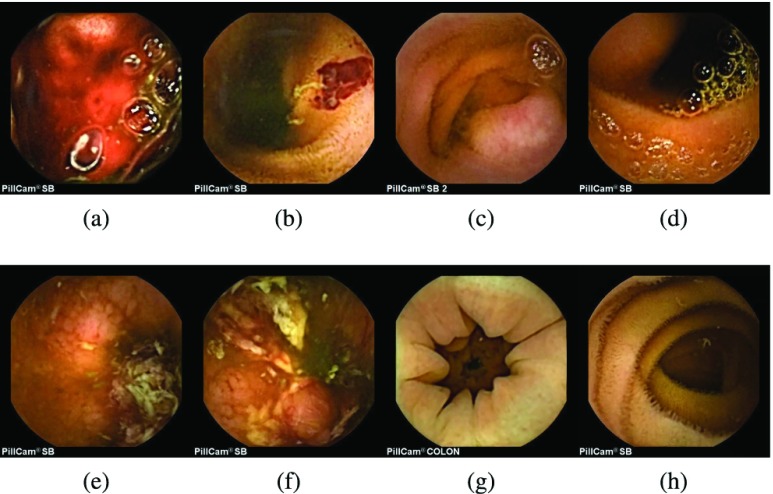
Typical WCE images; (a), (b) bleeding images; (c), (d) tumor images; (e), (f) ulcer images; (g), (h) WCE normal images.

**FIGURE 3.**
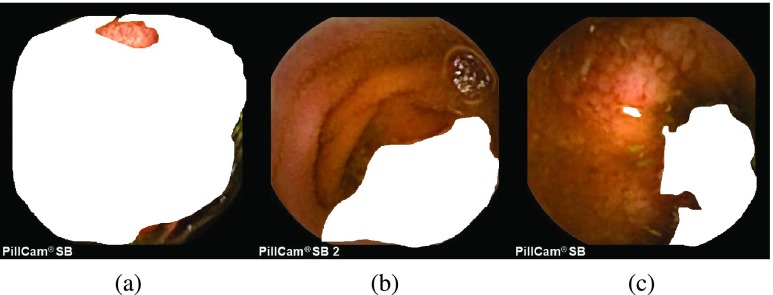
Pixel-labeled images of a sample (a) bleeding, (b) tumor and (c) ulcer image.

The block diagram of the proposed scheme for multi-disease detection is presented in Fig. 1. At first, with the help of given pixel-labeled images, multiple LDA models are trained. Later, the multiple trained LDA models are applied to WCE images to extract multiple ROIs followed by the PDF based feature extraction, and classification with a supervised hierarchical classifier. Typical examples of WCE bleeding, tumor, ulcer, and normal images are shown in Fig. 2. Fig. 2(a)-(b) represent the bleeding WCE image, whereas Fig. 2(c)-(d) represent the tumor images. Fig. 2(e)-(f) are ulcer images, whereas Fig. 2(g)-(h) are normal WCE images containing no abnormality. Fig. 3(a)–Fig. 3(c) present the pixel annotations of images in Fig. 2(a), 2(c), and 2(e) respectively, where the diseased portions are marked by the expert physicians.

### A. Multiple LDA (M-LDA) Model Generation From Pixel-Labeled Images

The proposed LDA model training scheme is described in this Section, which takes pixel-labeled diseased images as input and extracts the trained LDA models used later for obtaining the salient ROI from WCE images. Let us consider a single pixel-labeled diseased image marked by the expert physicians. Now, the pixels of pixel-labeled image are marked with 1 or 0 to form a binary image $Z \in \{0,1\}$, where the diseased pixels are represented by 1, and the normal pixels are represented by 0. As different types of diseases corresponds to different intensity patterns, we can implement LDA to train a model, which takes pixel intensities in diseased image as training input feature and their corresponding pixel-level annotations as the training labels. Thus, the trained model is able to learn the characteristic patterns of the diseased pixels present in a particular pixel-labeled image. As there can be many variants of the same disease, separate LDA models are trained to capture different variants of a particular disease. The choice behind using LDA is because LDA can be trained in real-time for a high number of trainer pixels with fairly high accuracy. Let, $^{k}\!I$ is the $k$-th RGB image where $R$, $G$ and $B$ represents the red, green and blue color channels and $(i,j)$ represents a coordinate. $^{k}\!Z$ represents the pixel-level binary annotation of image $^{k}\!I$. Therefore, the trained LDA model $mdl_{k}$ is described as

\begin{equation*} mdl_{k}= LDA~training(^{k}\!I,^{k}\!Z)\tag{1}\end{equation*}

An extracted LDA model, which is trained using a particular pixel-labeled image, can extract ROI in that image with a high precision. On the other side, the trained LDA model extracts ROI in a fairly well manner when applied to other images of same disease class. However, this model may not extract ROI with a high precision from images of other disease classes. Therefore, in order to successfully capture the ROI from a WCE image, multiple LDA (M-LDA) models are required, which are trained for capturing various salient patterns from different disease types. Let us assume, $\{^{k}\!I\}_{k=1}^{M}$ is the set of given pixel-labeled images with binary annotations $\{^{k}\!Z\}_{k=1}^{M}$. In this set, pixel-labeled images of each disease with different possible variants are present. After training the LDA models separately using all the pixel-labeled images, an LDA model bank $T$ is generated by grouping all the LDA models $T=\{mdl_{k}\}_{k=1}^{M}$.

### B. Salient ROI Extraction From M-LDA Models

Many works in literature extract features from the entire image for disease classification [7], [24]. In this case, the extracted feature quality degrades when the salient regions in an image is really small and image may get falsely classified. Moreover, in many cases, considering the entire image for feature extraction also results in an excess computation. Here, one possible way is to extract ROI before feature extraction. However, the major challenge here is to propose a unified scheme for ROI extraction of WCE images from any disease class, as different diseases show different characteristics in terms of their color patterns. For example, in [13], an ROI is extracted based on offline research for bleeding identification before computing features, where the ROI extraction parameters are static for all images. The static parameters may not extract ROI with significant precision when different variants of the same disease are available in the training set, even may not extract precise ROI from images of other diseases. On the other side, with the progress of WCE technology, the pixel-labeled images are available, where the salient regions are labeled by the experts. Nonetheless, this pixel-level knowledge is not utilized for image-level disease classification. Such knowledge is significant for capturing the disease characteristics from different classes. Hence, at first, the LDA models are trained for learning the disease characteristics from the prior pixel-labeled images. The resulting LDA models are then applied on WCE images for ROI extraction. As it cannot be determined which LDA model is best for ROI extraction from a WCE image, the proposed scheme extracts features from multiple ROIs which are captured by applying M-LDA models. This is why, M-LDA models were trained for learning the characteristics of different variants from different diseases. Let, an RGB image $J$ has red, green and blue color channels denoted by $R, G$ and $B$ respectively. Using the intensity values as features, the pixels in image $J$ are classified by applying the LDA model $mdl_{k}$ in the $predict$ function and the predicted labels are stored in $label_{k}$. For a model ${mdl_{k}} \in T$, the set of pixels ($S_{ROI,k}$) inside the ROI from image $J$ using $mdl_{k}$ are extracted as

\begin{equation*} S_{ROI,k}= \{(i,j)| label_{k}(i,j)=1 \}\tag{2}\end{equation*}

where

\begin{equation*} label_{k}(i,j)= predict(J(i,j), mdl_{k}); \quad \forall (i,j) \forall mdl_{k} \in T\tag{3}\end{equation*}

**FIGURE 4.**
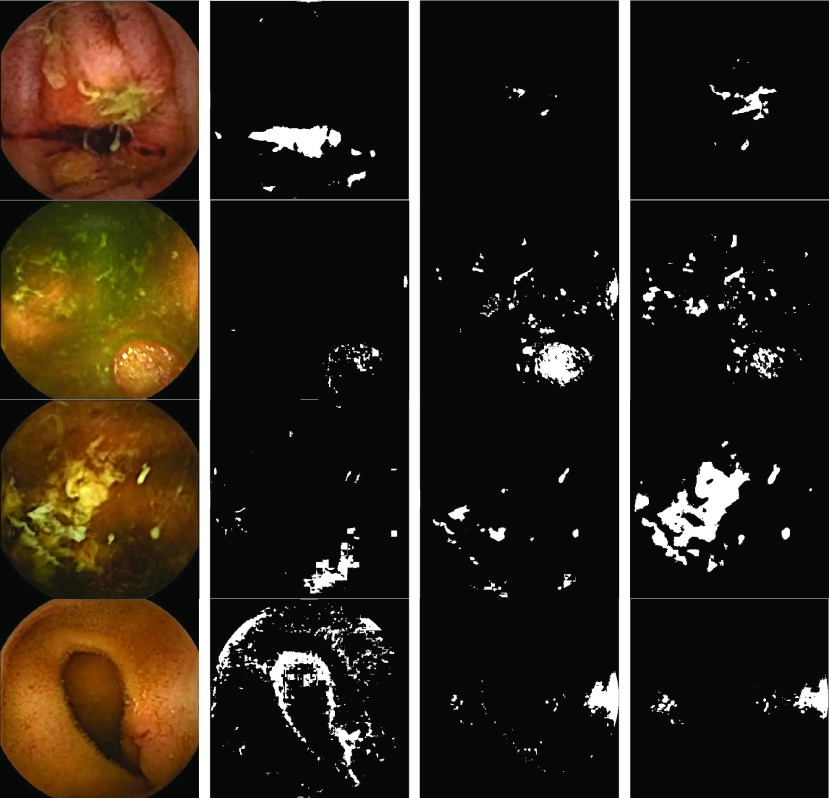
Extracted ROIs of bleeding, tumor, ulcer, and normal images using LDA models generated from other random pixel-labeled images. The figures in the first column represent RGB bleeding, ulcer, tumor and normal images respectively. The second, third and fourth columns represent the corresponding ROIs when random LDA models, generated respectively from other pixel-labeled bleeding, tumor and ulcer images, are applied. The extracted ROI is denoted by the white regions.

In $label_{k}$, it is expected that $S_{ROI,k}$ would be able to capture the desired ROI if ${mdl_{k}}$ is generated from a prior pixel-labeled image of the same disease class with similar variant. However, if ${mdl_{k}}$ is generated from an image of other disease type than the true class of the considered image, in most cases, only a few random pixels are expected to be in the $S_{ROI,k}$. In Fig. 4, sample bleeding, tumor and ulcer RGB images along with the extracted ROIs are presented. Here, the ROIs are extracted by applying various LDA models, such as models generated from other pixel-labeled images of the same disease class and of different disease classes. It is observed from the Figure that the LDA models can extract precise ROI when the models are generated from images of same disease class, whereas only a few random pixels are extracted in ROI when the models are generated from images of other disease classes. Hence, the extracted ROI does not confirm the class of a test image rather ROI only signifies that the extracted regions are probable salient regions. Moreover, the type of pixels extracted in $S_{ROI,k}$ depends on ${mdl_{k}}$. Therefore, to determine the class of the image, features are extracted from ROI.

### C. Salient Characteristic Pattern Extraction From ROI

**FIGURE 5.**
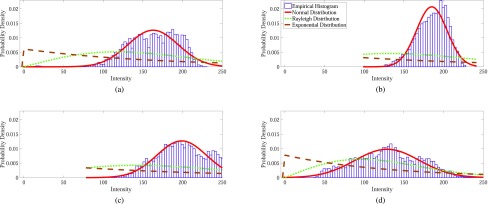
Model representations of salient intensity patterns of a sample (a) bleeding image; (b) tumor image; (c) ulcer image and (d) normal image. The salient pixels are extracted using a random LDA model. Along with the empirical histogram, all figures have 3 curves: fitted Normal PDF, Rayleigh PDF, and Exponential PDF to the ROI intensity pattern.

After capturing the set of pixels inside ROI $S_{ROI,k}$ by applying $mdl_{k}$ on image $J$, features are extracted using only the intensities of ROI. One possible idea, to capture the characteristic nature of ROI intensities in different color channels, is to fit the intensity patterns of ROI with a characteristic probability density function (PDF) model. In this way, the distribution of ROI intensities is explained with the help of fitted PDF parameters. Such parameterization of the intensity patterns of ROI provides the opportunity to learn the inherent characteristics of diseased patterns using only a few parameters. It is expected that in the fitted PDF, the class representations of different diseases will be reflected in a consistent manner. Therefore, the fitted PDF parameters can be treated as quality features with low feature dimension for disease classification which in turn, lead to a better classification performance. The model-fitting performance of various distributions is demonstrated with the help of Fig. 5. In Fig. 5, along with the empirical histogram, three PDFs, namely normal PDF, exponential PDF and Rayleigh PDF which are fitted on the ROI intensities at $R$ plane, are presented. In this case, the normal PDF fits the histogram better than other PDFs. Therefore, in this paper, a reasonable close PDF is fitted [31] suitable for modeling the ROI intensities at all color channels. The characteristic parameter(s) of the PDF are finally used as features, which involve reduced computational cost.

Let us assume, $\mathbf {x_{R_{k}}}$ is a vector consisting of the red ($R$) intensities $\forall (i,j) \in S_{ROI,k}$, where $S_{ROI,k}$ is extracted by applying the $k$-th LDA model ${mdl_{k}}~\in T$. $\sigma _{R_{k}}$ is the fitted parameter(s) when a PDF is fitted to $\mathbf {x_{R_{k}}}$. Similarly, all the LDA models in $T$ are applied separately in image $J$ for ROI extraction. Next, the ROI intensities for each color channel are separately fitted to a characteristic PDF. In order to acquire final feature vector ($\mathbf {F}$), all the fitted PDF parameters are concatenated as follows:

\begin{align*} \mathbf {f_{k}}=&\left[{\begin{array}{ccc}\sigma _{R_{k}} &\sigma _{G_{k}} &\sigma _{B_{k}} \end{array}}\right];\quad for~k=1,2, \ldots,M \tag{4}\\ \mathbf {F}=&\left[{\begin{array}{cccc}\mathbf {f_{1}} &\mathbf {f_{2}} &\ldots &\mathbf {f_{M}} \end{array}}\right]\tag{5}\end{align*}

Here, the feature dimension is directly proportional to the number of LDA models in $T$. However, if only a few number of pixel-labeled images are available for each disease type as a prior, the proposed scheme can be implemented using the fewer number of extracted LDA models.

### D. Hierarchical Classification Scheme

For the detection of GI diseases, in this paper, a hierarchical classification algorithm is tailored with the help of only a few binary SVM classifiers [32] as shown in Algorithm 1. To create non-linear decision functions, Gaussian radial basis function (RBF) kernel is employed, which maps the input space to a higher-dimensional feature space. Instead of implementing a hierarchical machine, one may consider a supervised classifier to perform the classification task considering a four-class problem using trainer for four classes, namely bleeding (‘$B$’), ulcer (‘$U$’), tumor (‘$T$’) and normal (‘$N$’). A major problem here is the imbalance among the members of different classes, which may not provide satisfactory performance, especially when the variation of class member number is very high. Generally, the number of normal class members is very high and hence, in the proposed method, first, a two-class supervised classification is employed considering ‘normal’ in one class and all other diseases in ‘non-normal’ or ‘diseased’ class (‘$\bar {N}$’). The $Machine$ function is a two-class SVM classifier, which takes the test feature, the two considered classes and the trainer-set with labels corresponding to two considered classes as input, and returns the predicted label of the test feature considering the two input classes. For example, $Machine$($F$, {‘$N$’,‘$\bar {N}$’}, $\mathcal {T}_{\{N, \bar {N}\}}$) takes the test feature $F$ as input and classifies $F$ either as ‘$N$’ or ‘$\bar {N}$’. Here, the trainer-set includes the normal examples in one class and the non-normal examples in another class. If output of the first machine is ‘non-normal’ (‘$\bar {N}$’), the input feature is sent in the second level of the algorithm to detect the class of disease present in the image. At this stage, in the second level, to classify a particular type of disease, again a two-class classifier is employed, where the trainer utilizes the members of a particular disease in one class and the remaining members in the other class. For example, to detect bleeding, a ‘bleeding versus non-bleeding’ (‘$B$ versus $\bar {B}$’) machine is employed. One possible way to confirm whether the test image has bleeding is to utilize only the prediction of ‘$B$ versus $\bar {B}$’ machine. However, instead of relying only on one machine, three separate machines are used to get a better decision accuracy. It is expected that if the ‘$B$ versus $\bar {B}$’ machine provides prediction as ‘$B$’, the other two machines will provide prediction as ‘$\bar {T}$’ in ‘$T$ versus $\bar {T}$’ machine and ‘$\bar {U}$’ in ‘$U$ versus $\bar {U}$’ machine. Although not expected, in the case, where two of the three machines (‘$B$ versus $\bar {B}$’, ‘$T$ versus $\bar {T}$’, and ‘$U$ versus $\bar {U}$’) provide positive results, another machine is required to confirm the disease type. For example, if the ‘$B$ versus $\bar {B}$’ machine declares the test label as ‘$T$’ and if the ‘$T$ versus $\bar {T}$’ machine declares the label as ‘$T$’ instead of ‘$\bar {T}$’, another stage is required to classify the image between ‘$B$’ and ‘$T$’. Similarly, when it comes ‘$U$’ instead of ‘$\bar {U}$’, further testing is performed to classify the image between ‘$B$’ and ‘$U$’. In this way, ulcer and tumor images are also classified. The major issues regarding the hierarchical classification scheme are

1) In the second level, instead of one machine, three separate machines are used.
2) To finalize the label of the test feature, decisions of all three machines at the second level are logically considered.

The machine parameters $C$ and $\sigma $ are optimized for each machine to achieve the best performance. In worst-case, the test feature goes through five binary machines.

**Algorithm 1:**
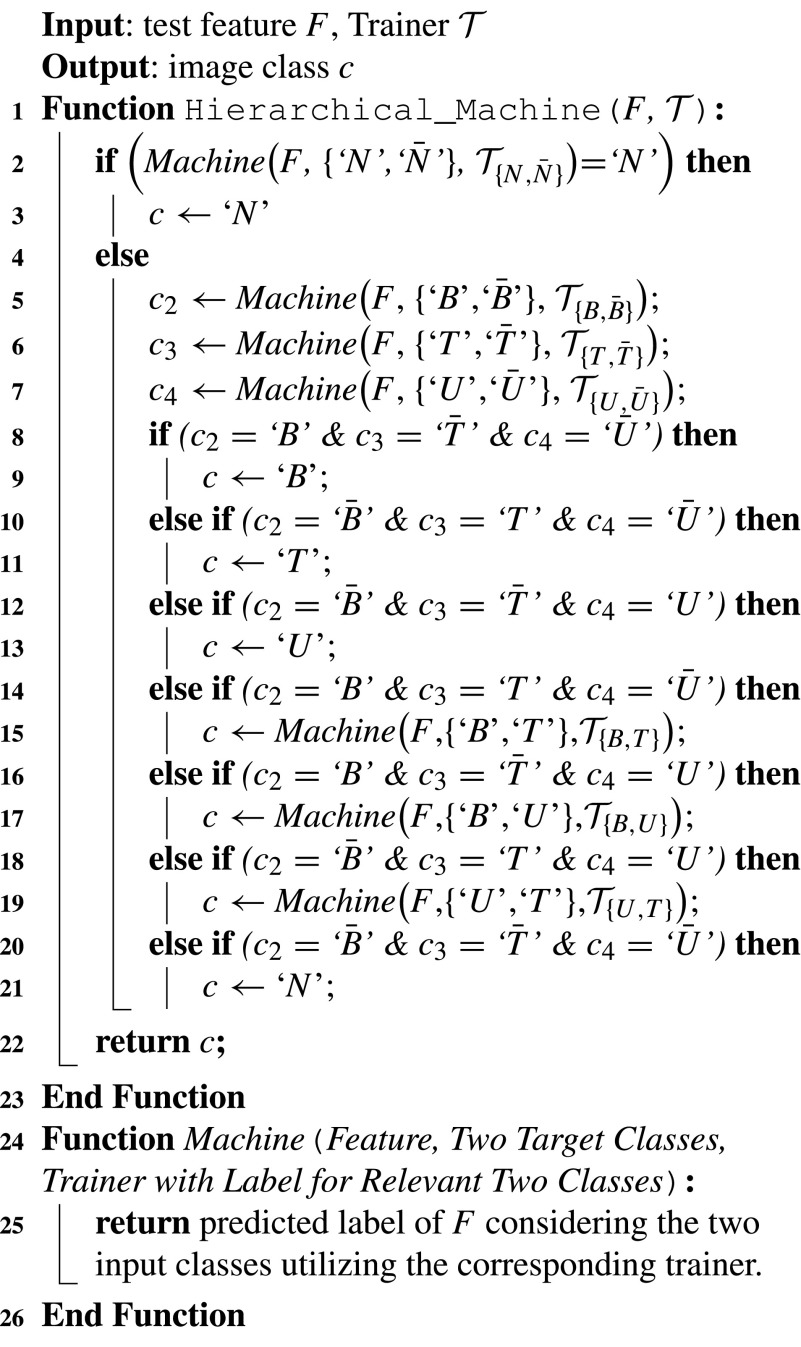
Algorithm for Hierarchical Classification

### E. Implementation for Single Disease Detection Problems

For dealing with single disease detection problems separately, such as the bleeding detection, the ulcer detection or the tumor detection, the proposed scheme can be reduced to a two class version. For example, while dealing with only bleeding detection problem, the LDA models are trained using only a set of prior pixel-labeled bleeding images. Later, only these LDA models are applied to extract the PDF based features from salient pixels. Moreover, for dealing with ‘Disease versus. Normal’ problem, the proposed scheme is implemented upto only the first level of Algorithm 1.

## III. Results and Discussion

In this Section, the experimental results obtained from the proposed method are presented. Along with results, data description and performance evaluation criteria are also presented. Ten-fold cross-validation scheme is used to evaluate the classification performance.

### A. Dataset and Performance Evaluation Criteria

For validation of the proposed scheme, the experiments are performed utilizing 50 WCE video clips accumulated from a widely used database in [33]. 15 of the 50 clips are bleeding clips, 10 are ulcer clips, 10 are tumor clips, and others are normal clips. In [33], only the clips are annotated by a disease type. For example, a clip is only marked as a bleeding video clip. The extracted images from a clip are not annotated. Moreover, each image inside a diseased video clip may not be a diseased image. Therefore, two expert physicians reviewed all the extracted images from all the clips to comment on them as bleeding, ulcer, tumor, or normal images. Experiments are carried out using all the images that are commented on as one of the four considered classes by the physicians. The total number of images is 2588, where 505 are bleeding images, 266 are ulcer images, 200 are tumor images, and 1617 are normal images. Moreover, for generating the pixel-labeled images, a good number of images from each disease type are selected randomly, as it is impossible to annotate all the images in pixel-level. Then, the images are marked by the physicians for creating pixel-level annotations. For extracting the M-LDA models, all the pixel-labeled images are considered. The total number of pixel-labeled bleeding, ulcer, and tumor images is 65, 31, and 30, respectively. In all images, the peripheral black pixels are discarded as a preprocessing step. The performance of multi-disease classification scheme is evaluated using standard measures, such as accuracy, precision, recall and F-1 score, whereas for reporting different two-class problems, performance measures, namely sensitivity, specificity, and accuracy are used.

### B. Multiple GI Disease Classification Performance

**FIGURE 6.**
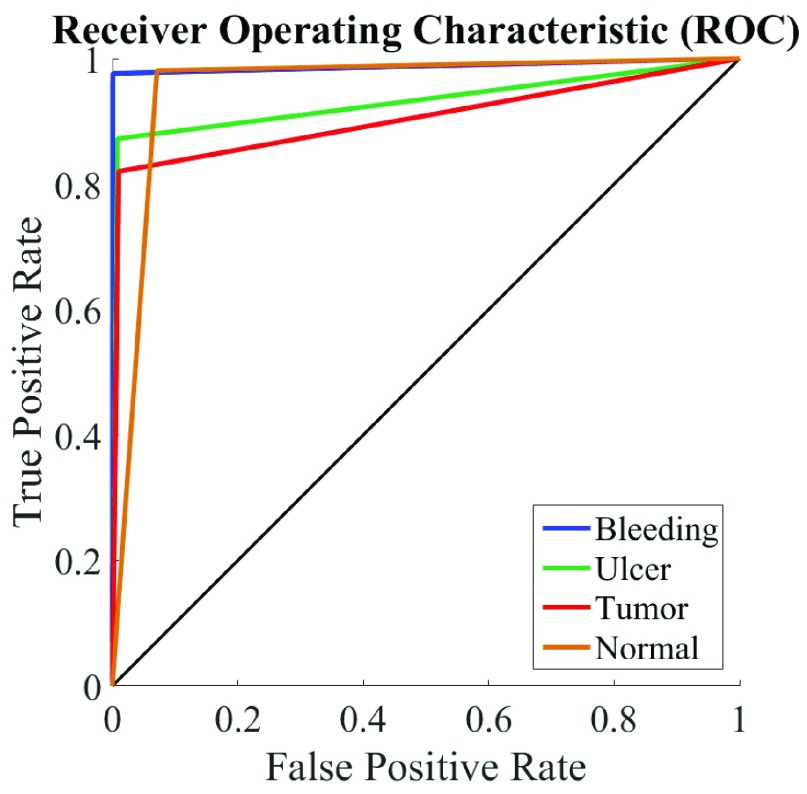
Receiver operating characteristic (ROC) curve for the proposed method. One ROC curve is drawn for each class as these curves are typically used in two-class problems.

In this Section, the performance of the proposed multi-disease detection scheme is demonstrated. In the proposed method, the LDA model training and the feature extraction are performed in RGB color space; for PDF fitting, the normal PDF is used and for classification, the hierarchical SVM classifier of Section II-D is implemented unless otherwise mentioned. The results obtained by the proposed method using the above mentioned settings are as follows: Accuracy is 91.38%, Precision is 87.14%, Recall is 85.41%, and F-1 score is 86.27%. To demonstrate how the proposed method performs in the midst of true and false classification, the receiver operating characteristics (ROC) curve for each class, using the settings mentioned above, is presented in Fig. 6. As each curve is close to the left and top edges of the plot, the classification performance is significantly good. The proposed method requires 0.9756 seconds per image for feature extraction and 0.00036575 seconds per image for testing using an Intel Core i-5 CPU @ 2.30GHz clock with 8.00GB RAM. All the computations are performed using MATLAB 2015b.

#### 1) Performance of the Proposed Scheme Using Different PDFS and Different Color Spaces

The results obtained by the proposed scheme using various color spaces, such as RGB, YIQ, LAB, HSV, YCbCr, and CMYK and using different distributions are demonstrated in Table 1. From the Table, it is observed that features from normal PDF in HSV space and YIQ space perform better than other PDFs in terms of all performance indices. As normal distribution performs better than other distributions in most cases, therefore, normal PDF is used in reporting the rest of the results.

**TABLE 1:** Performance (%) of the Proposed Scheme in Various Color Spaces Using Various PDFs

Color Space	PDF Model	Accuracy	Precision	Recall	F-1 score
RGB	Exponential	89.20	83.51	82.88	83.19
	Lognormal	87.75	82.61	80.11	81.34
	Nakagami	87.13	84.61	78.21	81.28
	Normal	91.38	87.14	85.41	86.27
	Poisson	89.21	84.76	83.83	84.29
	Rayleigh	89.31	85.33	82.31	83.79
	Rician	89.43	85.51	83.51	84.50
HSV	Exponential	92.71	90.14	88.03	89.10
	Lognormal	93.01	91.51	88.43	89.94
	Nakagami	91.21	88.53	83.27	85.81
	Normal	93.58	92.07	89.52	90.77
	Poisson	93.17	91.53	87.37	89.40
	Rayleigh	93.09	90.86	87.86	89.33
	Rician	92.52	91.91	87.06	89.41
YIQ	Exponential	90.68	89.29	83.53	86.31
	Lognormal	91.02	87.85	85.06	85.43
	Nakagami	88.13	88.19	79.53	83.63
	Normal	94.55	92.77	89.09	90.89
	Poisson	90.03	87.29	83.29	85.24
	Rayleigh	92.92	90.71	86.29	88.44
	Rician	92.17	91.34	85.29	88.21
YCbCr	Exponential	91.61	90.01	86.55	88.24
	Lognormal	92.11	90.28	86.53	88.37
	Nakagami	93.56	91.11	85.59	88.26
	Normal	93.00	91.23	87.58	89.36
	Poisson	92.56	89.99	87.69	88.82
	Rayleigh	91.55	90.00	84.12	86.96
	Rician	93.05	91.01	87.11	89.01
LAB	Exponential	90.21	87.11	83.56	85.29
	Lognormal	90.13	87.22	81.66	84.34
	Nakagami	91.21	90.01	85.01	87.43
	Normal	92.03	90.52	87.57	89.02
	Poisson	90.69	87.21	87.29	87.25
	Rayleigh	91.55	88.23	85.22	86.69
	Rician	91.56	90.56	84.01	87.16
CMYK	Exponential	91.01	87.58	83.12	85.29
	Lognormal	91.53	89.11	84.19	86.58
	Nakagami	91.03	90.01	82.28	86.01
	Normal	92.11	90.18	86.53	88.31
	Poisson	90.88	87.08	83.04	85.01
	Rayleigh	91.07	85.99	84.09	85.03
	Rician	92.11	91.55	84.63	87.95

#### 2) Performance Using Various Classifiers

Next, along with the hierarchical classifier in Section II-D, the proposed method is implemented with different supervised multi-class classifiers, such as K-nearest neighbors (KNN), LDA, Naive bayes (NB), artificial neural network (ANN), the error-correcting output codes (ECOC) model and the ensemble of classifiers. The KNN classifier is implemented using $K \in \{1,3,5,\ldots,11 \}$ with euclidean, cosine, cityblock and correlation distances, the ensemble of learners classifier is implemented with ‘AdaBoostM2’, ‘LPBoost’, ‘RUSBoost’, ‘TotalBoost’, and ‘Bag’ methods with ‘Discriminant’, and ‘Tree’ learners, the ANN classifier is implemented with scaled conjugate gradient back propagation, and gradient descent back propagation training function and the ‘crossentropy’ performance function with hidden layer size $N \in \{10, 20,\ldots, 80 \}$. The ECOC model is implemented using ‘one-versus-all’ and ‘one-versus-one’ coding scheme with SVM predictors using ‘linear’, ’polynomial’ and ‘RBF’ kernels. Besides, the hierarchical classifier mentioned in Section II-D is implemented with Gaussian RBF Kernel using $C \in \{2^{-2},2^{-1}, 2^{0}, \ldots, 2^{8}\}$ and $\sigma \in \{2^{-2},2^{-1}, 2^{0}, \ldots, 2^{8} \}$ in each binary classification stage. To obtain the best performance using the hierarchical classifier in Algorithm 1, the seven used $C$ values are 8, 4, 2, 4, 4, 128, and 32 and the seven $\sigma $ values are 32, 32, 32, 16, 16, 16, and 64 respectively. The best results of different machines are shown in Table 2 with the corresponding classifier settings. From the Table, one can observe that the best result obtained using the hierarchical SVM classifier of Section II-D outperforms the best result obtained from other classifiers.

**TABLE 2:** Comparison Among Different Classifiers

Classifier	Classifier Settings	Accuracy (%)	Precision (%)	Recall (%)	F-1 score (%)
KNN	K=3 with ‘cityblock’ distance	89.08	84.27	84.04	84.15
PNN	–	87.67	80.32	81.46	80.88
LDA	linear discriminant function	88.91	82.15	82.87	82.51
ANN	N=90 and scaled conjugate gradient back propagation training function	89.57	84.59	80.04	82.25
Ensemble of Learners	‘Bag’ method with ‘Tree’ learners	91.07	93.58	77.90	85.02
ECOC model	‘one-versus-one’ coding scheme with ‘polynomial’ SVM predictors	90.77	85.27	83.12	84.18
Hierarchical SVM	RBF Kernel	91.38	87.14	85.41	86.27

#### 3) Comparison With Other Variants of the Proposed Scheme

To demonstrate the performance of the proposed scheme, the method is implemented using several other variations than the proposed one. The results of the variant schemes are reported in Table 3.

1) Features from PDF Modeling Using the Entire Image: The proposed PDF modeling based feature extraction scheme is implemented using intensities from the entire image. From the results presented in Table 3, it is observed that using ROI intensities for PDF fitting performs better than to fit a PDF to pixel intensities of the entire image. Therefore, in this paper, ROI is extracted before feature extraction.
2) Count Features Instead of PDF Based Features: Instead of PDF fitting, one possible alternative is take the counts of pixels in the ROI set as features. It can be observed from Table 3 that the count based features performs poor than the proposed PDF based features in classifying the diseases.
3) Statistical Features Instead of PDF Based Features: Different statistical measures, such as mean, mode, median, etc. of the ROI intensities is chosen as features. It is observed from Table 3 that the statistical features cannot outperform the proposed PDF based features in classifying the diseases.
4) PDF fitting on Block Local Features Instead of ROI Intensities: The proposed method is implemented using the non-overlapping block-mean values as local features computed inside the ROI instead of PDF fitting on the ROI intensities. It can be observed from Table 3 that the PDF fitting of block local features cannot outperform the proposed scheme.
5) Proposed Method When Only 3 pixel-labeled Images Are Available for Each Disease: When the available number of pixel-labeled images is very small, the proposed scheme performs significantly well. To demonstrate this, only three pixel-labeled images are randomly chosen from each disease type for LDA model bank generation to implement the proposed scheme. From the results demonstrated in Table 3, it is observed that performance of the proposed scheme is significant in classifying the diseases even with the small amount of available pixel-labeled images.
6) Proposed Method using principal component analysis (PCA): In order to reduce the feature dimension, PCA is implemented after feature extraction. The classification results using the reduced feature are shown in Table 3. It is observed from the Table that the performance is reduced when the feature dimension is half compared to the original feature dimension.

**TABLE 3:** Performance Comparison of the Proposed Method With its Other Variants

Methods	Accuracy (%)	Precision (%)	Recall (%)	F-1 score (%)
1) PDF fitting on the entire image	86.51	78.99	78.67	78.83
2) Count features instead of the proposed PDF fitting based features	82.13	73.11	76.19	74.61
3) Statistical features instead of the proposed PDF fitting based features	88.00	85.12	81.39	83.21
4) PDF fitting on block local features inside ROI	82.28	78.23	76.29	77.21
5) Proposed method using 3 images from each disease type for Priori LDA model bank generation	89.23	86.13	83.13	84.60
6) Proposed method using PCA	89.93	86.32	80.52	83.32
7) Proposed Method	91.38	87.14	85.41	86.27

#### 4) Comparison Among Different State-of-the-Art Methods

Finally, the proposed method is compared with a few state-of-the-art methods available in [7]–[9], [24] and with the uniform local binary pattern (LBP) based scheme. The schemes are implemented using the data-set described in Section III-A and the classifier described in Section II-D. The results are reported in Table 4. Many single disease detection schemes [4], [15], [19] utilize LBP features. Therefore, for comparison, an uniform LBP histogram based feature is extracted to detect the GI diseases. Bag of words based methods are proposed in [7] for bleeding detection and in [24] for multiple GI disease detection. However, in this paper, for fair comparison, both methods are implemented for multiple GI disease detection. In [7], the vocabulary or the histogram bin centers are extracted by separately applying the K-means clustering algorithm (with $K=40$) on the pixel intensities of a few randomly chosen images from each disease types. On the other hand, in [24], a SALLC scheme is implemented to encode locally extracted features. However, instead of SIFT features, block statistical features are computed as local features. Method [7] is implemented using $YCbCr$ space whereas method [24] is implemented using $YIQ$ space as those color spaces are reported to perform better than others in the respective methods. In [9], block statistical features are combined from color planes for to create an indexed value to obtain a histogram to be utilized as features. In [8], a K-means clustering based scheme is employed in image blocks to obtain local statistical features from each cluster followed by the extraction of differential cluster features from the local block features. The compared methods use the entire image for feature extraction which degrade the detection performance for images having small diseased portion. From Table 4, it is observed that the results obtained from the proposed method outperforms the results obtained from other state-of-the-art methods in terms of all performance indices by a significant amount. Furthermore, the proposed method is compared with a convolutional neural network (CNN) based image classification scheme as CNN is becoming widely popular for image classification in recent times. The result is shown in Table 4. From the Table, it can be observed that the CNN based classification scheme cannot outperform the proposed method. The reason CNN based classification could not outperform the proposed method is that it perhaps requires a lot of images to train appropriately.

**TABLE 4:** Comparison Among Different Methods

Methods	Accuracy (%)	Precision (%)	Recall (%)	F-1 score (%)
Uniform LBP-histogram	86.69	83.11	78.99	81.00
Method [7]	88.05	86.32	81.39	83.78
Method [24]	90.35	86.12	85.07	85.59
Method [8]	87.32	86.45	81.11	83.69
Method [9]	88.49	87.34	80.87	83.98
2-Dimensional CNN	88.51	85.41	83.27	84.32
Proposed method using $RGB$ space	91.38	87.14	85.41	86.27
Proposed method using $YIQ$ space	94.55	92.77	89.09	90.89

In the proposed method, the feature dimension depends on the number of available pixel-labeled images. Hence, to vary the feature dimension, a total number of $p \in \{10, 20, 30, \ldots, 120\}$ pixel-labeled images are chosen randomly from all the pixel-labeled images to generate the M-LDA models. The reduced set of pixel-labeled images include images from all disease classes. The best result, considering the maximum F-1 score and the minimum feature dimension, is achieved for a set of randomly chosen 10 pixel-labeled images, which corresponds to a feature dimension of 60. The accuracy, precision, recall, and F-1 score using this setting are 90.89%, 87.01%, 85.13%, and 86.06%, respectively. Moreover, the M-LDA models are trained using the pixel-labeled images of the considered diseases, which posses the pixel-level knowledge of the diseases. Next, the trained LDA models are applied to both the training and test images to select the possible diseased portions (POI) before feature extraction. Therefore, if we could incorporate the pixel-labeled images of other disease classes in the trainer, it is expected that the LDA models would capture the POI of those diseases. Hence, it is expected that the proposed method should do well (if extended) in classifying further disease categories.

### C. Performance of Two Class Classification Problems

Table 5 presents the classification performance of the proposed method using binary SVM classifier using Gaussian RBF kernel in different two class problems, such as ‘bleeding versus normal’, ‘ulcer versus normal’ and the best performance in each case is reported in Table 5. Moreover, besides the SVM classifier, various other supervised classifiers, such as LDA, NB, KNN, ANN and ensemble of classifiers are also implemented with different settings to evaluate the performance of the proposed method in two class problems. The best results with settings in each case are reported in Table 6. It is observed from Table 5 and Table 6 that, in most cases, the best result of the SVM classifier outperforms the other classifiers’ best results.

**TABLE 5:** Binary Classification Results Using SVM With RBF Kernel

Problem	Sen. (%)	Spe. (%)	Acc. (%)
Bleeding versus Normal	95.96	97.88	97.39
Ulcer versus Normal	90.17	92.63	92.17
Tumor versus Normal	87.10	91.95	91.45
Bleeding versus Ulcer	96.04	96.27	96.09
Bleeding versus Tumor	96.01	97.52	97.15
Ulcer versus Tumor	93.23	93.34	93.30
Disease versus Normal	92.56	93.18	92.90
Bleeding versus Other	94.25	96.27	95.79
Ulcer versus Other	90.11	90.26	90.20
Tumor versus Other	86.04	85.16	85.32

**TABLE 6:** Performance of Two Class Problems Using Different Settings of Various Classifiers Except the Binary SVM Classifier

Methods	KNN classifier, with Cityblock Distance			Ensemble of learners classifier with Discriminant Learner and ‘RUSBoost’ method			ANN classifier with N= 40 and training function ’trainscg’		
Problem	Sen. (%)	Spe. (%)	Acc. (%)	Sen. (%)	Spe. (%)	Acc. (%)	Sen. (%)	Spe. (%)	Acc. (%)
Bleeding versus Normal	94.93	97.00	96.29	94.43	95.17	94.81	91.51	96.68	95.67
Ulcer versus Normal	85.23	95.33	93.14	84.99	92.43	90.24	72.81	96.76	93.24
Tumor versus Normal	71.11	94.21	91.74	71.27	91.01	89.17	79.15	97.04	92.31
Bleeding versus Ulcer	96.12	94.41	95.53	91.73	91.80	91.67	95.73	91.80	91.23
Bleeding versus Tumor	97.15	95.99	97.31	95.13	91.05	94.34	97.05	92.29	95.23
Ulcer versus Tumor	94.14	90.23	92.58	86.11	78.58	82.12	90.14	87.94	88.91
Disease versus Normal	90.15	90.21	90.19	85.16	88.23	88.09	80.43	91.79	86.98
Bleeding versus Other	91.23	97.12	91.16	90.13	94.14	93.12	86.81	97.07	94.17
Ulcer versus Other	85.01	96.12	95.12	83.25	91.22	90.01	72.52	97.01	93.54
Tumor versus Other	71.11	96.64	94.59	72.13	86.82	86.13	75.13	98.11	93.51

### D. Performance in Continuous Clips

Performance of the proposed scheme is reported using 15 continuous video clips. Here, the used performance indices are sensitivity, specificity and accuracy. The same pixel-labeled images are used for obtaining the trained LDA models. The results are presented in Table 7. Specificity is undefined for Clip 2, 3 and 7 as they only contain diseased images of the corresponding class. Satisfactory performance of the proposed method in continuous video clips indicates that the proposed method can be implemented to help the physicians in reviewing WCE videos with significant accuracy.

**TABLE 7:** Performance in Continuous Videos (%)

Video Type	Clip No	Accuracy	Sensitivity	Specificity
bleeding	1	90.00	87.50	90.48
bleeding	2	94.00	94.00	–
bleeding	3	96.00	96.00	–
bleeding	4	87.00	82.22	90.91
bleeding	5	87.00	75.00	90.00
ulcer	6	90.00	87.50	90.22
ulcer	7	95.00	95.00	–
ulcer	8	90.00	70.00	92.22
ulcer	9	94.00	100.00	93.81
ulcer	10	90.00	88.24	100.00
tumor	11	84.00	75.00	85.71
tumor	12	91.00	80.00	94.67
tumor	13	92.00	89.58	94.23
tumor	14	98.37	89.36	99.48
tumor	15	85.00	80.00	85.26

## IV. Conclusion

An automatic multiple GI disease detection scheme is proposed in this paper based on PDF modeling of multiple LDA classified ROI. First, the ROI from given WCE images are extracted by applying the proposed LDA based scheme. Next, a characteristic PDF model is fitted on the pixel intensity patterns inside the ROI to utilize the PDF parameters as features in the classifier. Generally, pixel-labeled images are available only in small number which, in most cases, are not at all utilized for detecting diseases from WCE images. The main concept here is to learn the disease characteristics from the pixel-level knowledge by training LDA models on pixel-labeled images which are later used for obtaining ROI in WCE images both in training and test phases. From the experiments, it is found that extracting features from ROI provides better feature quality even though the salient region is small compared to the features computed inside the entire image. PDF fitting to the pixel intensity patterns inside the ROI can capture the characteristic nature of diseases using only few parameters and can also reduce the feature dimension significantly. For classification, a hierarchical SVM classifier is employed in this paper utilizing only a few two-class SVMs, which is found fruitful in detecting the GI diseases. It is experimentally shown that the results obtained using normal PDF fitted on intensity patterns of ROI in $YIQ$ space outperforms the results obtained using other PDFs in all other color spaces. Moreover, it is also found that the proposed method is able to perform significantly well even if the number of available prior pixel-labeled images is small. Finally, the goodness of the proposed method is ensured when a significantly well performance in continuous WCE videos is obtained. Therefore, the proposed multiple GI disease detection scheme is expected to help physicians in reviewing a large number of WCE images to diagnose different GI diseases.
